# Foetal onset of *EIF2B* related disorder in two siblings: cerebellar hypoplasia with absent Bergmann glia and severe hypomyelination

**DOI:** 10.1186/s40478-020-00929-2

**Published:** 2020-04-15

**Authors:** Aurélien Trimouille, Florent Marguet, Fanny Sauvestre, Eulalie Lasseaux, Fanny Pelluard, Marie-Laure Martin-Négrier, Claudio Plaisant, Caroline Rooryck, Didier Lacombe, Benoît Arveiler, Odile Boespflug-Tanguy, Sophie Naudion, Annie Laquerrière

**Affiliations:** 1 Department of Medical Genetics, Bordeaux University Hospital, Bordeaux, France; 2 Bordeaux Univ, INSERM U1211, Bordeaux University Hospital, Bordeaux, France; 3 Normandie Univ, UNIROUEN, INSERM U1245, Rouen University Hospital, Department of Pathology, F76000 Rouen, France; 4 Department of Pathology, Bordeaux University Hospital, Bordeaux, France; 5 INSERM UMR1141 Paris Diderot University, APHP Child neurology and Metabolic Department, Reference Centre for Leukodystrophies (LEUKOFRANCE –ERN-RND)– Robert-Debré hospital, Paris, France; 6 Pathology Laboratory, Pavillon Jacques Delarue,CHR, 1 rue de Germont, 76031 Rouen, Cedex France

**Keywords:** Vanishing white matter disease, Foetal neuropathology, Whole exome sequencing, Diagnosis, Pathophysiology

## Abstract

Bi-allelic pathogenic variants in genes of the EIF2B family are responsible for Childhood Ataxia with Central nervous system Hypomyelination/Vanishing White Matter disease, a progressive neurodegenerative disorder of the central white matter. Only seven molecularly proven cases with antenatal onset have been reported so far. We report for the first time the neuropathological findings obtained from two foetuses harbouring deleterious variants in the *EIF2B5* gene who presented in utero growth retardation and microcephaly with simplified gyral pattern that led to a medical termination of the pregnancy at 27 and 32 weeks of gestation. Neuropathological examination confirmed microcephaly with delayed gyration, periventricular pseudo-cysts and severe cerebellar hypoplasia. Histologically, the cerebellar cortex was immature, the dentate nuclei were fragmented and myelin stains revealed almost no myelination of the infratentorial structures. Bergmann glia was virtually absent associated to a drastic decreased number of mature astrocytes in the cerebellar white matter, multiple nestin-positive immature astrocytes as well as increased numbers of PDGRFα-positive oligodendrocyte precursors. Whole exome sequencing performed in the two foetuses and their parents allowed the identification of two *EIF2B5* compound heterozygous variants in the two foetuses: c.468C > G p.Ile156Met and c.1165G > A p.Val389Met, the parents being heterozygous carriers. These variants are absent in the genome Aggregation Database (gnomAD r2.0.2). Contrary to the variant Ile156Met already described in a patient with CACH syndrome, the variant p.Val389Met is novel and predicted to be deleterious using several softwares. Neuropathological findings further expand the phenotypic spectrum of the disease that very likely occurs during early gestation and may manifest from the second half of pregnancy by a severe impairment of cerebral and cerebellar development.

## Introduction

Childhood Ataxia with Central nervous system Hypomyelination/Vanishing White Matter disease (VWMD) (MIM#603896) is one of the most prevalent autosomal recessive lethal leukodystrophy. VWMD was initially described in children who presented with normal early neurological development followed by progressive decline with episodes of rapid deterioration caused by trauma, acute fright or fever associated with typical magnetic resonance imaging (MRI) features [1]. It is caused by germline deleterious variants in any of the genes encoding the five subunits of the evolutionary conserved eukaryotic initiation factor 2 eIF2B, a protein complex involved in the regulation of mRNA translation [2]. Currently, a large phenotypic variation has been reported from antenatal life to late adulthood, the severity of the prognosis being inversely correlated with age of onset [3]. In infants, children and adults, the morphological hallmarks associate white matter rarefaction with cystic degeneration without any or with only subtle microglial reaction, decreased number of mature oligodendrocytes often displaying a foamy pattern and poor reactive gliosis with the presence of dysmorphic and immature astrocytes that makes the disease fall into the group of astrocytopathies [4]. We report herein for the first time autopsy findings in two foetal siblings interrupted at 27 and 32 weeks of gestation (WG) for microcephaly with cerebellar hypoplasia, which harbored two compound heterozygous pathogenic variants in the *EIF2B5* gene, one of the two being novel.

## Case presentation

In a thirty-seven-year-old woman, gravida 2 para 0, routine ultrasound (US) performed at 22 WG revealed severe intrauterine growth restriction and cerebral anomalies consisting of microcephaly, vermis hypoplasia and bilateral hypoechogenic zones close to the anterior horns of the lateral ventricles for which a diagnosis of porencephaly was suggested. Due to unfavorable prognosis, a medical termination of the pregnancy (TOP) was achieved at 27 WG. Four months later, the parents conceived a second child. At 22 WG, foetal growth parameters were within the normal range, but US examination at 30 WG showed severe growth restriction and recurrence of the brain lesions leading to TOP at 32 WG. Array-CGH was normal in both foetuses, as was the karyotype (46, XX and 46, XY respectively). The unrelated parents had no personal or familial medical history.

**Fig. 1:**
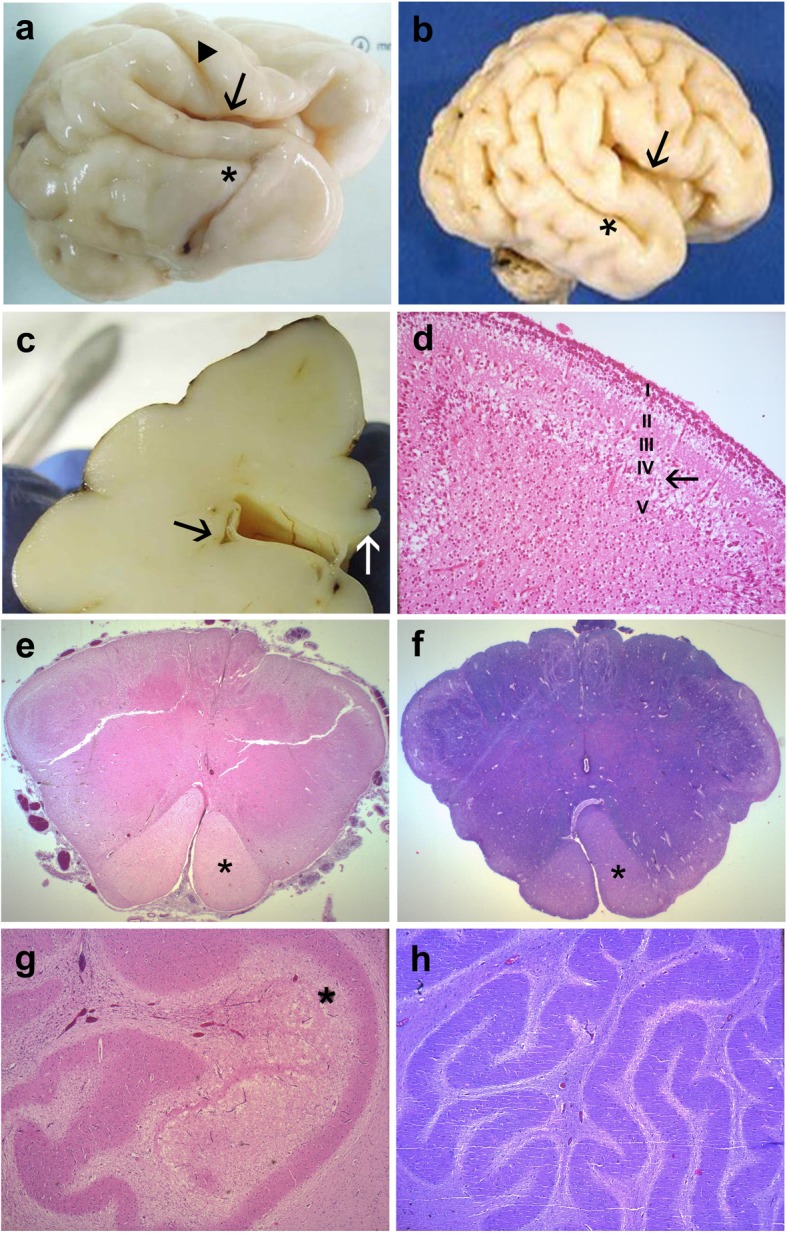
Main macroscopic and histological findings of *EIF2B5* mutated foetal brain aged 32 WG. **a** External view of the right hemisphere of the second foetus showing almost closed Sylvian fissure (arrow), absent postcentral gyrus whereas the central fissure is present (arrowhead) and dysmorphic superior temporal gyrus (asterisk) with poor secondary sulcation. **b** compared with an age-matched control brain where the Sylvian fissure is still open (arrow), with all primary and secondary sulci and gyri already formed, in particular the superior temporal sulcus (asterisk). **c** With on coronal section passing through the diencephalon where several small-sized periventricular pseudo-cysts are observed (black arrow) whereas the corpus callosum is normal (white arrow). **d** Histology of the cerebellar cortex displaying an abnormal five-layered immature cortex with abnormal persistence of the lamina dissecans (transient layer IV, arrow) corresponding to a developmental stage of 24 WG [OM × 100]. **e** Luxol- phloxin stain of the medulla oblongata just above the decussation of pyramids (asterisk) displaying no myelin in the second foetus [OM × 16]. **f** Compared with an age matched control, where all fascicles are myelinated, except corticospinal tracts (asterisk) which begin to be myelinated [OM × 16]. **g** Absent myelination around the poorly convoluted dentate nucleus and vacuolization of the cerebellar white matter inside the dentate nucleus (asterisk) [OM × 50]. **h** Compared with the control where the dentate nucleus is normally convoluted and surrounded by myelinated white matter [OM × 50]

A complete autopsy was carried out in both foetuses according to standardized protocols with the informed written consent of the parents in accordance with the French law and after approval by our local ethical committee (Supplementary material S1 Methods with additional references). Foetal weights were below the 3rd percentile. Neither craniofacial particularities nor macroscopical and histological visceral abnormalities were noted. In the first foetus, histological examination of the ovaries did not reveal any ovarian dysgenesis and the density of primordial follicles appeared to be similar to what observed in two 27 WG control ovaries. Brain weight was 88 g in the first foetus and 153 g in the second foetus. Infratentorial weights were 4.3 g and 6 g, corresponding to a developmental age of 22 and 26 WG respectively, in favour of microcephaly with cerebellar hypoplasia. In the second foetus, brain gyration was delayed with almost no secondary sulci (Fig. 1a and b). Cerebellar foliation was also delayed, the tertiary folia being still absent. On coronal sections, periventricular pseudo-cysts were observed within the ganglionic eminences close to the caudate nuclei (Fig. 1c). Cerebellar abnormalities consisted in poorly convoluted dentate nuclei and a five-layered cerebellar cortex in the two foetuses (Fig. 1d). Luxol-phloxin stains were entirely negative in the brainstem of the second foetus (Fig. 1e) compared with an age-matched control brain (Fig. 1f). No pycnotic or foamy oligodendrocytes were observed within and around the dentate nuclei (Fig. 1g and h). At the supratentorial level, no gaps in the glia limitans were observed. The cortical plate was depleted in neurons mainly in layer III but no microcolumnar dysplasia was noted, arguing against a disruption of cortical plate cytoarchitectony which could result from impaired migration or neuron positioning. In the second case, the pyramidal morphology of neurons in layers III and V was not apparent, indicative of immaturity as pyramidal morphology is easily recognizable 26 WG onward. In the second case, histological examination of the eyes was normal using routine stainings.

**Fig. 2:**
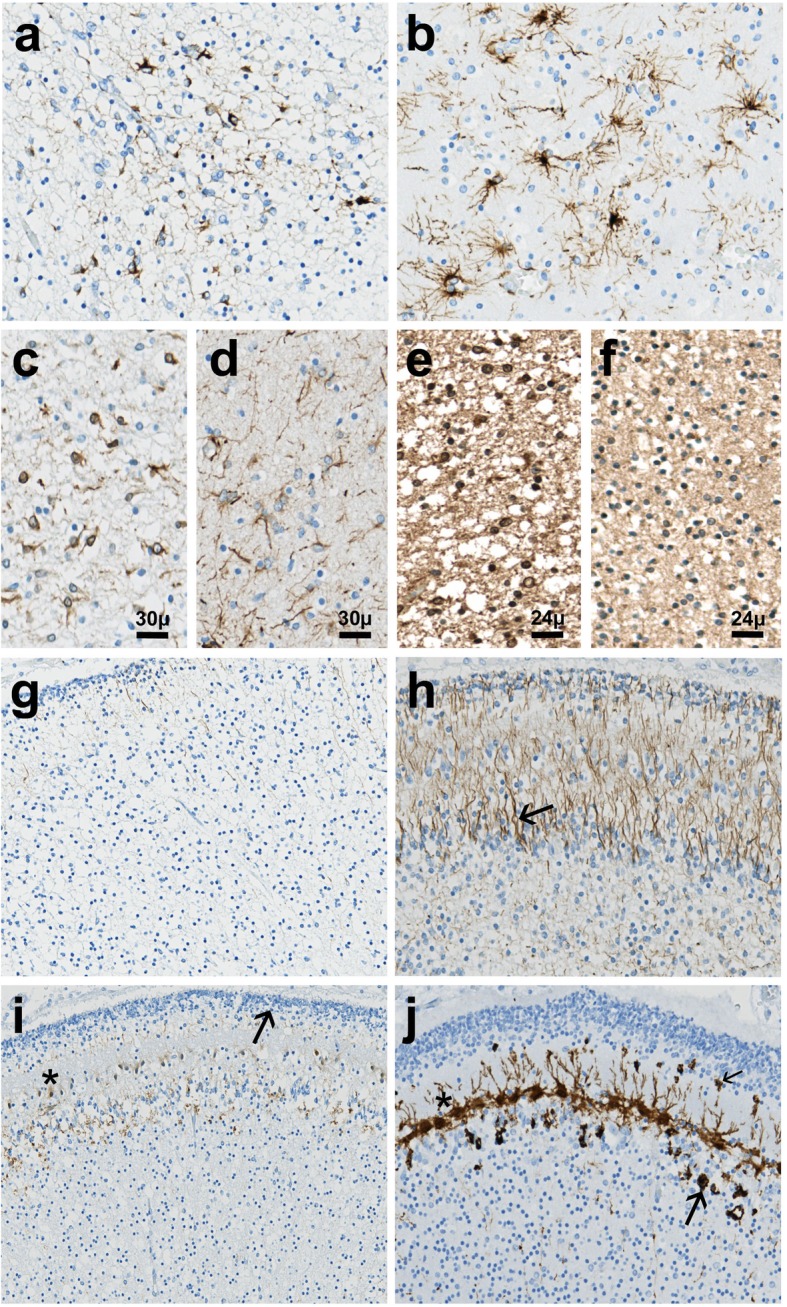
Immunohistochemical findings in the cerebellum of the second affected foetus. **a** GFAP immunolabeling of the cerebellar white matter showing immature astrocytes possessing coarse or thin and short cell processes [OM × 400]. **b** Contrary to the control cerebellar white matter which contains numerous mature astrocytes with multiple fine processes [OM × 400]. **c** Nestin immunostaining demonstrating overexpression of this intermediate filament in immature astrocytic cell somata [OM × 400]. **d** Contrary to control cerebellar white matter in which nestin expression is mainly restricted to some astrocytic processes [OM × 400]. **e** Intense PDGFRα immunoreactivity of the oligodendrocyte precursors in the patient’s cerebellar white matter [OM × 400]. **f** Contrasting with almost absent PDGFRα expression in the control cerebellar white matter [OM × 400]. **g** Virtually absent GFAP-immunoreactivity of the Bergmann glia in the patient’s cerebellar cortex [OM × 200]. **h** Compared with the control cerebellar cortex in which Bergmann cells are located adjacent to Purkinje neurons (arrow), observed throughout the molecular layer up to the transient external granular cell layer [OM × 200]. **i** Patient’s cerebellar cortex exhibiting weak calbindin immunoreactivity in the small immature and dysmorphic Purkinje cells devoid of apical dendritic arborizations (asterisk), forming a discontinuous layer [OM × 100]. **j** Whereas in control cerebellum, Purkinje cell somata and dendritic trees (asterisk) are strongly calbindin-immunoreactive as are stellate cells (thin arrow) and Golgi II neurons (thick arrow) [OM × 100]. OM: original magnification

Vimentin immunohistochemistry did not make it possible to find radial glia or glia limitans abnormalities, and GFAP immunolabelings revealed some accumulation of reactive astrocytes around the periventricular cavities. But whatever the location, i.e., surrounding the cavities or at a distance from them, astrocyte morphology was similar. No differences in shape or number of GFAP-positive Muller cells between the affected foetus and an age matched control eye were observed. Conversely, it displayed a decreased number of mature astrocytes in the cerebellar white matter. Their severely altered morphology consisted of underdeveloped processes compared with an age-matched control (Fig. 2a and b). Their cytoplasms exhibited no alpha-B crystallin accumulation. Immunolabelings using the intermediate filament nestin, a marker of radial glial cells confirmed the presence of numerous immature astrocytes by comparison with the control brain (Fig. 2c and d). Even though Olig2 immunolabeling revealed no significant differences pre-oligodendrocyte number, an increased number of PDGFRα-positive oligodendrocyte precursors was observed (Fig. 2e and f). Bergmann glia was virtually absent (Fig. 2g and h). Calbindin immunoreactivity was weak in Purkinje cells, the density of which was reduced with almost no associated immunolabelling of Golgi II neurons and stellate cells (Fig. 2i and j).

Whole Exome Sequencing (WES) (detailed procedures in Supplementary material S1 Methods with additional references and S2 Missense variant considered in this study), allowed for the identification of two compound heterozygous missense variants in the *EIF2B5* gene (NM_003907.2) in the two foetuses: c.468C > G p.Ile156Met and c.1165G > A p.Val389Met. Each parent was heterozygous for one of the two variants which are absent in the GnomAD, ESP and in the dbSNP databases. Contrary to the deleterious p.Ile156Met variant which has already been reported in a patient with VWMD [5], the p.Val389Met is novel. This variant has a CADD score of 29.6 and is predicted as damaging using SIFT, Mutation Taster and Polyphen-2 prediction tools [6]. No deleterious variants were found in the other currently known genes involved in other leukodystrophies.

## Discussion and conclusion

To date, only seven *EIF2B* mutated patients from four unrelated families affected by congenital form of VWMD with antenatal onset have been reported [3, 7]. Antenatal symptoms consisted in severe growth restriction, oligohydramnios and decreased foetal movements or arthrogryposis. Microcephaly was present at birth in one neonate whereas progressive cerebellar atrophy and delayed gyration appeared later on MRI in all cases. In some patients with early disease onset, involvement of other organs has been reported such as hepatosplenomegaly, pancreatic abnormalities, kidney hypoplasia, ovary dysgenesis resulting in premature ovarian failure and cataracts [8]. Intriguingly, no visceral lesions were identified in the two foetuses at autopsy. Furthermore, no cataracts were present and GFAP immunohistochemistry did not reveal any lesion of Muller glia. Neuropathological examination performed in two affected infants with antenatal onset aged 8 and 10 months disclosed severe white matter loss in the cerebral hemispheres, pons, cerebellum and spinal cord with a diffuse decrease in oligodendrocyte number and no reactive gliosis. As in our cases, periventricular pseudo-cysts were reported [8, 9]. In a neuropathological review of affected children by Bugiani et al. in 2018, white matter rarefaction usually appears after birth but cystic degeneration of the deep white matter appears later, contrary to our cases in which cavitated lesions were present from the second trimester [10]. Histologically, an increased number of oligodendrocytes in the hemispheric white matter despite myelin paucity has been reported in children’s or adult brains [11] conversely to infants’ brains in which mature oligodendrocyte numbers appear to be normal, a finding that could not be confirmed in both foetuses as mature, myelinating oligodendrocytes are mostly produced 30 WG onward. Nevertheless, immunohistochemical studies revealed that at 32 WG the cerebellar white matter mainly contained oligodendrocyte precursors instead of maturing oligodendrocytes, resulting rather from delayed than defective oligodendrocyte maturation, in so far as normal numbers of mature oligodendrocytes have been reported from birth to childhood [4, 9, 10]. Immunohistochemical analyses also revealed severe astrocytic lesions in the cerebellar white matter. Dysmorphic astrocytes presented severely altered morphologies consisting in smaller size, thin or coarse and short processes. Moreover, they still expressed nestin, indicating that they remained at an astroglial progenitor stage, as previously shown in a mouse model by Dooves et al. [12, 13]. Such alterations have also been recently described using patient-derived induced pluripotent stem cells (iPSCs). VWMD iPSCs-derived astrocytes expressed nestin as in the present cases, and the mean length of the longest processes was diminished by a factor of 2. Astrocytes also overexpressed alpha-B-crystallin contrary to foetal astrocytes in which no accumulation was visualized, an event that probably occurs after birth [4, 14]. Besides, it has been shown in several previous studies that astrocytes influence oligodendrocyte differentiation and maturation. In a recent study on human and mouse VWM iPSCs, Leferink et al. demonstrated that the white matter astrocytic subpopulation is selectively vulnerable to VWM mutations and that oligodendrocyte precursor cell maturation is inhibited by VWM astrocytes [15]. These findings very likely explain the over-representation of PDGFRα-positive oligodendrocyte precursors observed in our cases, resulting from impaired astrocyte differentiation and functioning. Moreover, it has been shown that in older VWMD affected patients, Bergmann glial somata are translocated to the molecular layer whereas they should be located close to Purkinje cells, this translocation representing a hallmark of VWMD for Dooves et al. [13, 16]. This disease marker has also been reported in a spontaneous mutant mouse in the *Eif2b5* gene called the “toy mouse” [17]. In the two foetuses, virtually absent Bergmann glia somata and processes suggested a defect of Bergmann glia development and/or maintenance from early stages of gestation. Even if a specific granular neuron loss could not be evaluated in our cases as these cells are mainly generated from the 30th WG to 13 post-natal months, the transient external granular cell layer as well as Purkinje cell and internal granular cell layers were poorly developed with almost absent stellate and Golgi II cells, suggesting that EIF2B5 is not only required for astrocyte and oligodendrocyte generation and differentiation [18] but also for the production, migration and survival of cerebellar neurons resulting in cerebellar hypoplasia.

**Fig. 3:**
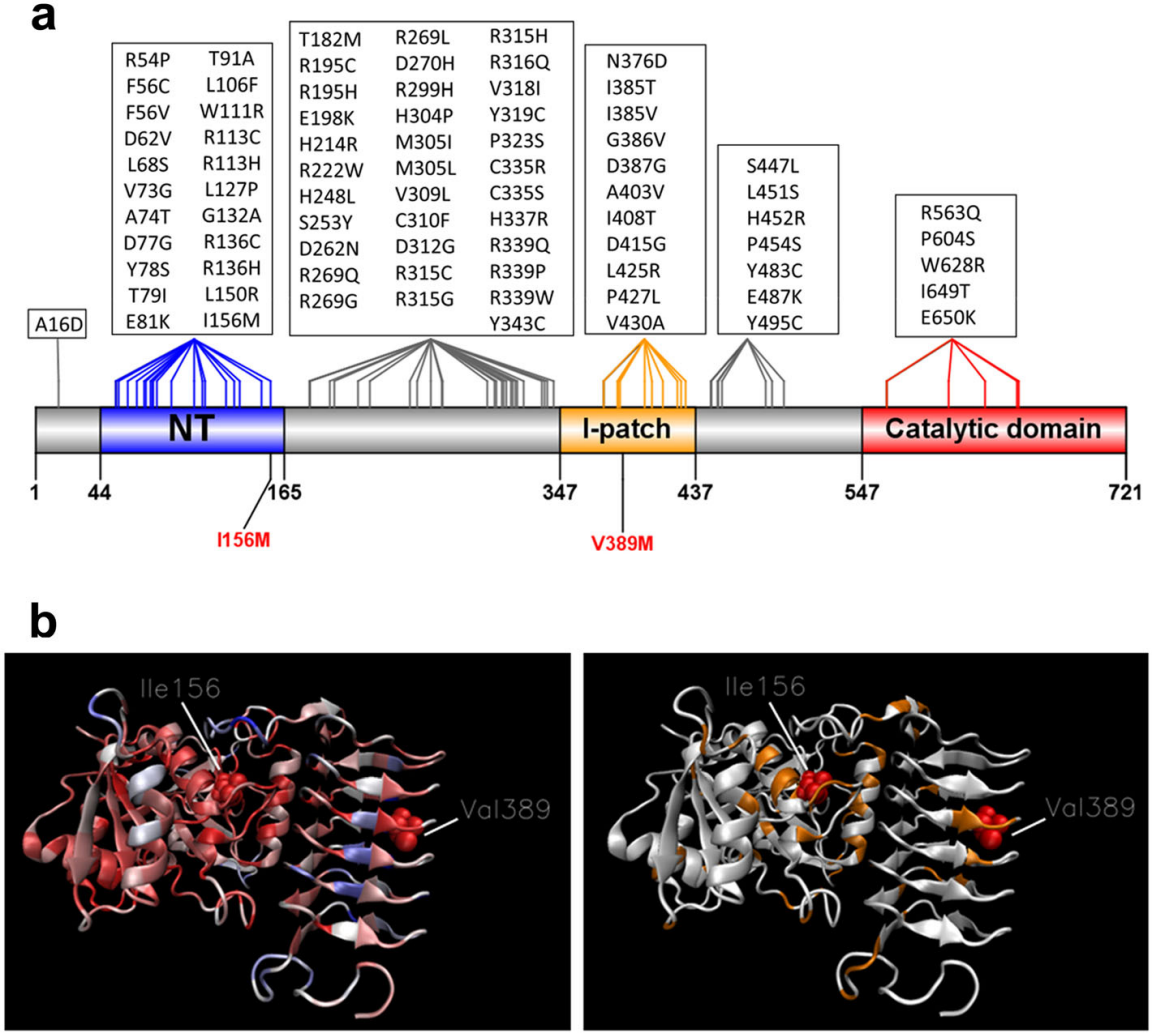
Molecular findings in EIF2B related disorders. **a** Schematic representation of the previously reported deleterious missense variants in the literature, and those identified in our cases (in red). **b** 3D Model of EIF2B5 protein. Left: Consurf score of residues (red: conserved; blue: not conserved). p.156 and p.389 are shown with Van der Waals representation. Right: Positions of the pathogenic variants (yellow)

More than 80 different pathogenic amino acid changes of *EIF2B5* on 67 different positions have been reported in patients with VWMD (Fig. 3a) according to the HGMD Pro [7] and Clinvar databases [19] (Supplementary information S2). The average normalized Consurf conservation score on 150 species of the amino acids affected by pathogenic variants is − 0,434, that means that mutated residues are more conserved than other EIF2B5 residues. However, as previously reported, the range of scores (min − 1421, max 1563) indicates that several mutated residues are not conserved [16, 20]. *EIF2B5* variants are located across various domains of the protein with no clear hotspot. Consurf analysis shows that the p.Ile156 and p.Val389 amino acids which belong to different domains are conserved, with scores of − 0.918 and − 1.069 respectively (Fig. 3b). Concerning the p.Ile156 amino-acid, even if this position may be occupied depending on species by an Isoleucine or a Valine, two very similar hydrophobic amino acids (Grantham score: 29), the p.Ile156 may be considered as conserved. Besides, genotype-phenotype correlation studies have shown that in EIF2B related disorders, the severity of the disease directly depends on the nature of the *EIF2B5* deleterious variants [21]. From these observations, it could be speculated that the disease severity observed in our foetal cases may be due to a specific effect of the two variants, especially the newly described p.Val389Met variant. But one cannot exclude that the extremely severe phenotype observed in our two siblings may be related to the impact of not yet identified modifier genes or of critical post-transcriptional regulators such as miRNAs. It could also be hypothesized that additional variants in other genes not detected using WES or random developmental processes involving other cellular pathways could be responsible for the phenotypic severity.

In conclusion**,** neuropathology findings together molecular data reported here further expand the phenotypic spectrum of VWMD which may occur early during gestation and may manifest from the second part of the pregnancy by defective Bergmann glia responsible for severe cerebellar hypoplasia.

## Supplementary information

**Additional file 1.**

**Additional file 2.**

## Data Availability

All data generated or analyzed during this study are included in this article and its supplementary files.

## Abbreviations

iPSCs: Induced pluripotent stem cells
MRI: Magnetic resonance imaging
NGS: Next-Generation Sequencing
TOP: Termination of the pregnancy
US: Ultrasound
VWM: Vanishing white matter
VWMD: Vanishing white matter disease
WES: Whole-Exome Sequencing
WG: Weeks of gestation
