# Supplementary material for: Foetal onset of EIF2B related disorder in two siblings: cerebellar hypoplasia with absent Bergmann glia and severe hypomyelination
Source: Acta Neuropathol Commun. 2020 Apr 15;8:48. doi: 10.1186/s40478-020-00929-2 (PMC7161274; doi:10.1186/s40478-020-00929-2)
Supplement: Supplementary file 1 — Additional file 1. [file 40478_2020_929_MOESM1_ESM.doc]

**Supplementary material: Methods with additional references**

**Morphological studies**

Autopsy procedures were performed according to standardized protocols including X-rays, photographs, macroscopical and microscopical examination of all viscerae. The brains were fixed in a 10% formalin-zinc buffer solution for one month. Brain maturation and biometric data were evaluated according to Fees Higgins Clarke and Larroche and to Guihard-Costa and Larroche [1; 2]. Multiple seven-micrometer sections obtained from paraffin-embedded tissue were stained using haematoxylin-eosin. As myelination starts during fetal life around birth in the cerebral hemispheres and from 27 WG in the brainstem and cerebellum, Luxol-phloxin stains were performed on infratentorial sections from the brain of the second fetus. Routine immunohistochemistry was carried out according to standardized protocols using antibodies directed against glial fibrillary acidic protein (GFAP, 1:300; Dakopatts, Trappes France), αB-crystallin (1:500; Millipore, Molsheim, France), nestin (1:500; Millipore, Olig2 (1:200; Epitomics, Burlingame, CA), PDGFRα (1:50; Thermo scientific, Fremont, CA), and calbindin (1:100; Tebu Novo-castra- Le Perray en Yvelines- France).

1. Feess-Higgins A, Larroche JC (1987) Development of the human fetal brain. (Masson eds) Paris, INSERM CNRS.

2. Guihard-Costa AM, Larroche JC. Differential growth between the fetal brain and its infratentorial part. Early Hum Dev. 1990;23:27–40.

**Molecular studies**

Genomic DNA was extracted from fetal tissues and from peripheral blood lymphocytes of both parents. Whole-Exome Sequencing (WES) was performed in both fetuses and their parents. Library preparation, exome capture, sequencing and data analysis were carried out by means of IntegraGen SA (Evry, France).

Sequence capture, enrichment and elution were performed according to manufacturer’s instruction and protocols using SureSelect XT Clinical Research Exome in-solution enrichment methodology (Agilent, Santa Clara, California) without modification except for library preparation performed with NEBNext® Ultra II kit (New England Biolabs®). For library preparation 200 ng of each genomic DNA were fragmented by sonication and purified to yield fragments of 150-200 bp. Paired-end adaptor oligonucleotides from the NEB kit were ligated on repaired, were then hybridized to the SureSelect oligo probe capture library for 72 hr. After hybridization, washing, and elution, the eluted fraction was PCR-amplified with 9 cycles, purified and quantified by QPCR to obtain sufficient DNA template for downstream applications. Each eluted-enriched DNA sample was then sequenced on an Illumina HiSeq4000 (Illumina, San Diego, California) as Paired End 75 reads. Image analysis and base calling is performed using Illumina Real Time Analysis (2.7.7) with default parameters.

Sequence reads were mapped to the Human genome build (hg38) by using the Burrows-Wheeler Aligner (BWA) tool. The duplicated reads were removed (sambamba tools). Variant calling was performed via the Broad Institute’s GATK Haplotype Caller GVCF tool (3.7). Annotation and variants filtering were performed using Cartagenia Lab Bench NGS platform (Agilent Technologies). Only rare variants (allele frequency <1% and absence of homozygotes in GnomAD database), with a potential effect on proteins (non-synonym, and synonym or intronic variants within 5 bp of a splice site), or already described in the HGMD database (Qiagen) were kept for analysis.

Three-D model

The 6CAJ protein model [3] was visualized using VMD software [2], a tool developed with NIH support by the Theoretical and Computational Biophysics group at the Beckman Institute, University of Illinois at Urbana-Champaign. Consurf analysis of the 6CAJ model was performed using ConSurf providing multiple sequence alignment on 150 species with standard parameters (available upon request) [1].

1. Ashkenazy H, Abadi S, Martz E, Chay O, Mayrose I, Pupko T, et al. ConSurf 2016: an improved methodology to estimate and visualize evolutionary conservation in macromolecules. Nucleic Acids Res. 2016;44:W344-350.

2. Humphrey W, Dalke A, Schulten K. VMD: Visual molecular dynamics. J Mol Graph. 1996;14:33–8.

3. Tsai JC, Miller-Vedam LE, Anand AA, Jaishankar P, Nguyen HC, Renslo AR, et al. Structure of the nucleotide exchange factor eIF2B reveals mechanism of memory-enhancing molecule. Science. 2018;359:eaaq0939.
