# Supplementary material for: Foetal onset of EIF2B related disorder in two siblings: cerebellar hypoplasia with absent Bergmann glia and severe hypomyelination
Source: Acta Neuropathol Commun. 2020 Apr 15;8:48. doi: 10.1186/s40478-020-00929-2 (PMC7161274; doi:10.1186/s40478-020-00929-2)
Supplement: Supplementary file 2 — Additional file 2. [file 40478_2020_929_MOESM2_ESM.doc]

| Missense variants considered in this study | |  |  |  |
| --- | --- | --- | --- | --- |
| assembly : GRCh37 |  |  |  |  |
| transcript : NM_003907.2 | |  |  |  |
| protein : NP_003898.2 |  |  |  |  |
| g. nomenclature | c. nomenclature | p. nomenclature | dbSNP ID | Clinvar ID |
| g.183853220C>A | c.47C>A | p.(Ala16Asp) | rs113994041 |  |
| g.183853334G>C | c.161G>C | p.(Arg54Pro) | rs113994042 |  |
| g.183853340T>G | c.167T>G | p.(Phe56Cys) | rs121908541 | RCV000006316.5 |
| g.183853339T>G | c.166T>G | p.(Phe56Val) | rs113994043 | RCV000006315.5 |
| g.183853358A>T | c.185A>T | p.(Asp62Val) |  |  |
| g.183854407T>C | c.203T>C | p.(Leu68Ser) | rs113994044 |  |
| g.183854422T>G | c.218T>G | p.(Val73Gly) | rs113994045 |  |
| g.183854424G>A | c.220G>A | p.(Ala74Thr) | rs113994046 |  |
| g.183854434A>G | c.230A>G | p.(Asp77Gly) |  |  |
| g.183854437A>C | c.233A>C | p.(Tyr78Ser) |  |  |
| g.183854440C>T | c.236C>T | p.(Thr79Ile) |  |  |
| g.183854445G>A | c.241G>A | p.(Glu81Lys) | rs113994047 | RCV000281339.1 |
| g.183854475A>G | c.271A>G | p.(Thr91Ala) | rs28939717 | RCV000255738.1|RCV000006305.5 |
| g.183854522A>T | c.318A>T | p.(Leu106Phe) | rs113994048 | RCV000724783.1|RCV000175759.2|RCV000624816.1 |
| g.183855418T>C | c.331T>C | p.(Trp111Arg) |  |  |
| g.183855424C>T | c.337C>T | p.(Arg113Cys) | rs113994050 |  |
| g.183855425G>A | c.338G>A | p.(Arg113His) | rs113994049 | RCV000254893.4|RCV000006309.3|RCV000006308.7 |
| g.183855467T>C | c.380T>C | p.(Leu127Pro) | rs1057521084 | RCV000439475.1 |
| g.183855482G>C | c.395G>C | p.(Gly132Ala) |  |  |
| g.183855493C>T | c.406C>T | p.(Arg136Cys) | rs113994051 |  |
| g.183855494G>A | c.407G>A | p.(Arg136His) | rs958193703 |  |
| g.183855536T>G | c.449T>G | p.(Leu150Arg) |  |  |
| g.183855555C>G | c.468C>G | p.(Ile156Met) |  |  |
| g.183855724C>T | c.545C>T | p.(Thr182Met) | rs113994053 | RCV000416181.1|RCV000006313.4 |
| g.183855762C>T | c.583C>T | p.(Arg195Cys) | rs113994055 | RCV000006312.3 |
| g.183855763G>A | c.584G>A | p.(Arg195His) | rs113994054 | RCV000006310.4 |
| g.183855771G>A | c.592G>A | p.(Glu198Lys) | rs2971410 |  |
| g.183855820A>G | c.641A>G | p.(His214Arg) |  |  |
| g.183855843C>T | c.664C>T | p.(Arg222Trp) | rs151061485 |  |
| g.183856012A>T | c.743A>T | p.(His248Leu) |  |  |
| g.183856027C>A | c.758C>A | p.(Ser253Tyr) |  |  |
| g.183857886G>A | c.784G>A | p.(Asp262Asn) |  |  |
| g.183857908G>A | c.806G>A | p.(Arg269Gln) | rs113994057 |  |
| g.183857907C>G | c.805C>G | p.(Arg269Gly) | rs113994058 |  |
| g.183857908G>T | c.806G>T | p.(Arg269Leu) |  |  |
| g.183857910G>C | c.808G>C | p.(Asp270His) | rs397514646 | RCV000033200.4 |
| g.183858258G>A | c.896G>A | p.(Arg299His) | rs113994060 | RCV000412998.1 |
| g.183858273A>C | c.911A>C | p.(His304Pro) |  |  |
| g.183858277G>A | c.915G>A | p.(Met305Ile) | rs1431803321 |  |
| g.183858275A>T | c.913A>T | p.(Met305Leu) | rs200143780 |  |
| g.183858287G>C | c.925G>C | p.(Val309Leu) | rs113994061 | RCV000006311.4 |
| g.183858291G>T | c.929G>T | p.(Cys310Phe) | rs113994062 |  |
| g.183858297A>G | c.935A>G | p.(Asp312Gly) |  |  |
| g.183858305C>T | c.943C>T | p.(Arg315Cys) | rs113994063 | RCV000735327.1 |
| g.183858305C>G | c.943C>G | p.(Arg315Gly) |  |  |
| g.183858306G>A | c.944G>A | p.(Arg315His) | rs113994064 | RCV000006314.3 |
| g.183858309G>A | c.947G>A | p.(Arg316Gln) | rs766187010 |  |
| g.183858314G>A | c.952G>A | p.(Val318Ile) |  |  |
| g.183858318A>G | c.956A>G | p.(Tyr319Cys) | rs759376966 |  |
| g.183858329C>T | c.967C>T | p.(Pro323Ser) | rs113994066 |  |
| g.183858365T>C | c.1003T>C | p.(Cys335Arg) | rs113994067 |  |
| g.183858366G>C | c.1004G>C | p.(Cys335Ser) |  |  |
| g.183858372A>G | c.1010A>G | p.(His337Arg) |  | RCV000722142.1 |
| g.183858378G>A | c.1016G>A | p.(Arg339Gln) | rs113994069 | RCV000421811.1 |
| g.183858378G>C | c.1016G>C | p.(Arg339Pro) | rs113994069 |  |
| g.183858377C>T | c.1015C>T | p.(Arg339Trp) | rs113994068 |  |
| g.183858390A>G | c.1028A>G | p.(Tyr343Cys) | rs113994072 |  |
| g.183858488A>G | c.1126A>G | p.(Asn376Asp) |  |  |
| g.183858516T>C | c.1154T>C | p.(Ile385Thr) |  |  |
| g.183858515A>G | c.1153A>G | p.(Ile385Val) | rs113994073 |  |
| g.183859713G>T | c.1157G>T | p.(Gly386Val) | rs113994074 | RCV000006307.4 |
| g.183859716A>G | c.1160A>G | p.(Asp387Gly) | rs113994075 |  |
| g.183859764C>T | c.1208C>T | p.(Ala403Val) | rs545593935 |  |
| g.183859779T>C | c.1223T>C | p.(Ile408Thr) |  |  |
| g.183859800A>G | c.1244A>G | p.(Asp415Gly) |  |  |
| g.183859830T>G | c.1274T>G | p.(Leu425Arg) | rs113994077 |  |
| g.183859836C>T | c.1280C>T | p.(Pro427Leu) | rs113994078 |  |
| g.183859845T>C | c.1289T>C | p.(Val430Ala) | rs113994079 |  |
| g.183860062C>T | c.1340C>T | p.(Ser447Leu) | rs113994080 |  |
| g.183860074T>C | c.1352T>C | p.(Leu451Ser) |  |  |
| g.183860077A>G | c.1355A>G | p.(His452Arg) |  |  |
| g.183860082C>T | c.1360C>T | p.(Pro454Ser) | rs766921114 |  |
| g.183860293A>G | c.1448A>G | p.(Tyr483Cys) | rs758301189 |  |
| g.183860304G>A | c.1459G>A | p.(Glu487Lys) | rs113994081 |  |
| g.183860329A>G | c.1484A>G | p.(Tyr495Cys) | rs113994082 |  |
| g.183860873G>A | c.1688G>A | p.(Arg563Gln) | rs1290134782 |  |
| g.183861294C>T | c.1810C>T | p.(Pro604Ser) |  |  |
| g.183861899T>C | c.1882T>C | p.(Trp628Arg) | rs28937596 | RCV000006306.3 |
| g.183861963T>C | c.1946T>C | p.(Ile649Thr) | rs1064794256 | RCV000763510.1|RCV000483052.1 |
| g.183861965G>A | c.1948G>A | p.(Glu650Lys) | rs113994085 |  |
